# Entrapped central venous catheter guide wire

**DOI:** 10.4103/0019-5049.68378

**Published:** 2010

**Authors:** Sarika Katiyar, Rajnish Kumar Jain

**Affiliations:** Department of Anesthesiology and Critical Care, Bhopal Memorial Hospital and Research Centre, Bhopal - 462 038, India

Sir,

The use of central venous catheterisation is common in the setting of ICU for monitoring of CVP, fluid administration and drug infusions. In our unit by current estimates, 1,700 catheters are inserted per year. However, placement of CVCs is not innocuous. Numerous complications may occur, with varying frequency and severity. Acute complications like: arrhythmias, arterial puncture, pneumothorax, etc., are readily apparent. Rarer complications such as those involving guide wire insertion or removal often are not readily apparent. Most common complication of the passage of a guide wire is the induction of cardiac arrhythmias. Other complications include: looping, knotting, vascular perforation, fragmentation and embolisation, entrapment of the wire in the IVC filter and the Sternocleidomastoid muscle. We describe the entrapment of guide wire probably in the subcutaneous tissue.

A 35-year-old female presented in emergency with tachypnoea and hypotension. She was a known case of Chronic Renal Failure since five years and was on regular dialysis. On ABG analysis: her PO_2_ was 68 mm Hg, pH-7.36, pCO_2_ -21mm Hg SpO_2_ was 89%. Her USG was done which revealed bilateral pleural effusion. Other physical examination findings were within normal limit.

She was shifted to ICU for further management and dialysis. She was put on noninvasive ventilation which she tolerated well. In view of haemodynamic instability central venous cannulation was planned. After ensuring appropriate coagulation status, right-sided internal jugular vein was chosen for cannulation. A seven French percutaneous Seldinger type 15 cms length catheter was used. Under all aseptic precautions right internal jugular vein was cannulated with 24-gauge needle followed by introducer needle which punctured the vein on first attempt.

**Figure 1:**
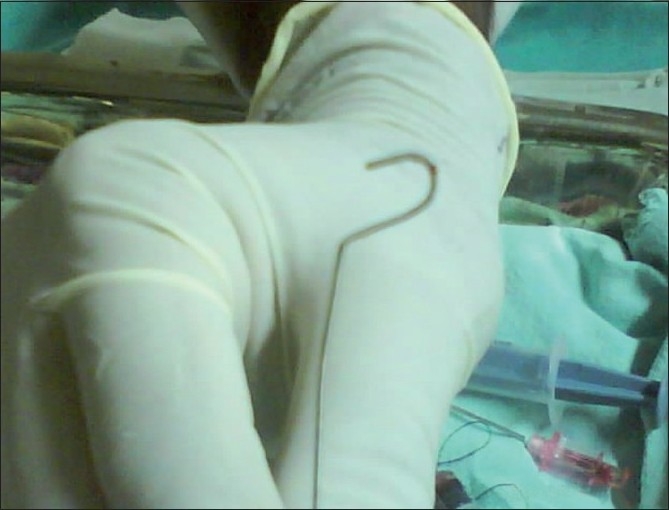
Tip of the CVC guide wire after removal

After aspiration of blood, guide wire was introduced, but it could not be introduced more than five centimeters. Thus, we tried to remove the guide wire, to reconfirm the backflow of blood in the introducer needle. But guide wire was stuck and many attempts to remove it with gentle traction failed. During this traction the introducer needle slipped and only the guide wire was left in place. We got a portable Chest X-ray done, which revealed that only J-tip of guide wire was stuck. Thus, after excluding guide wire knotting removal of guide wire was attempted again and it came out with gentle traction [Figure 1]. After removal of guide wire local pressure was applied. USG was done to reveal any injury or local haematoma. Gradually, patient improved and was shifted to ward.

Central Venous Catheterisation placement is an extremely common procedure performed at virtually every institution by a variety of specialists. Central Venous Catheterisation is traditionally done blindly by skin landmarks. However, National Institute for Clinical Excellence (NICE) has published its recommendations for use of ultrasound locating devices for placing central venous catheters. Ultrasound reduces the incidence of complications related to venous puncture.[1,2] Complications can still occur after the safe venous puncture including, the guide wire, the dilator or the catheter insertion complication.

Catheter related complications are well known, but there are very few reports in which guide wire has been involved. Most common guide wire related complications include entrapment of guide wire in the Sternocleidomastoid muscle and Inferior Venacava Filters.[3–5] Most of the reports published mentions initial difficulty with the passage of guide wire. Any resistance felt during guide wire insertion should prompt needle withdrawal several millimeters. If the guide wire cannot be passed easily without resistance, procedure should be stopped; needle and guide wire should be removed as a single unit and pressure applied. In our case the needle was withdrawn and guide wire pulled thereafter. Probably, the J-tip of guide wire got stuck just underneath the skin. Luckily, in this case guide wire could be easily removed in few attempts of gentle traction; otherwise it would have required surgical exploration.

Here we want to emphasize that even though central venous catheter placement is common, execution of details is imperative. We should be aware of potential entrapment of guide wire during central venous catheterisation. CVCs often are placed by personnel who are in training; closer supervision by more senior person may help identify and prevent similar complications. It is also important that force should never be used to withdraw a guide wire when unexpected resistance is encountered, as this may result in fracture of the wire and damage to the internal structures.
